# Homozygosity Haplotype and Whole-Exome Sequencing Analysis to Identify Potentially Functional Rare Variants Involved in Multiple Sclerosis among Sardinian Families

**DOI:** 10.3390/cimb43030125

**Published:** 2021-10-27

**Authors:** Teresa Fazia, Daria Marzanati, Anna Laura Carotenuto, Ashley Beecham, Athena Hadjixenofontos, Jacob L. McCauley, Valeria Saddi, Marialuisa Piras, Luisa Bernardinelli, Davide Gentilini

**Affiliations:** 1 Department of Brain and Behavioral Sciences, University of Pavia, 27100 Pavia, Italy; daria.marzanati01@universitadipavia.it (D.M.); annala.carotenuto@gmail.com (A.L.C.); luisa.bernardinelli@unipv.it (L.B.); davide.gentilini@unipv.it (D.G.); 2 John P. Hussman Institute for Human Genomics, Miller School of Medicine, University of Miami, Miami, FL 33136, USA; ABeecham@med.miami.edu (A.B.); hadjixenofontos@gmail.com (A.H.); jmccauley@med.miami.edu (J.L.M.); 3 Dr. John T. Macdonald Foundation Department of Human Genetics, Miller School of Medicine, Miami, FL 33136, USA; 4 Divisione di Neurologia, Presidio Ospedaliero S. Francesco, ASL Numero 3 Nuoro, 08100 Nuoro, Italy; Valeria.saddi@tircali.it (V.S.); marialpiras@tiscali.it (M.P.); 5 Bioinformatics and Statistical Genomics Unit, Istituto Auxologico Italiano IRCCS, 20095 Cusano Milanino, Italy

**Keywords:** multiplex families, Sardinian population, WES data, rare variants, low-frequency variants, Homozygosity Haplotype analysis, multiple sclerosis, Region form Common Ancestor (RCA)

## Abstract

Multiple Sclerosis (MS) is a complex multifactorial autoimmune disease, whose sex- and age-adjusted prevalence in Sardinia (Italy) is among the highest worldwide. To date, 233 loci were associated with MS and almost 20% of risk heritability is attributable to common genetic variants, but many low-frequency and rare variants remain to be discovered. Here, we aimed to contribute to the understanding of the genetic basis of MS by investigating potentially functional rare variants. To this end, we analyzed thirteen multiplex Sardinian families with Immunochip genotyping data. For five families, Whole Exome Sequencing (WES) data were also available. Firstly, we performed a non-parametric Homozygosity Haplotype analysis for identifying the Region from Common Ancestor (RCA). Then, on these potential disease-linked RCA, we searched for the presence of rare variants shared by the affected individuals by analyzing WES data. We found: (*i*) a variant (43181034 T > G) in the splicing region on exon 27 of *CUL9*; (*ii*) a variant (50245517 A > C) in the splicing region on exon 16 of *ATP9A*; (*iii*) a non-synonymous variant (43223539 A > C), on exon 9 of *TTBK1*; (*iv*) a non-synonymous variant (42976917 A > C) on exon 9 of *PPP2R5D*; and *v*) a variant (109859349-109859354) in 3′UTR of *MYO16.*

## 1. Introduction

Multiple Sclerosis (MS) is a complex neurological autoimmune disease, which mainly affects people in early adulthood; for this reason, it is considered as the most common cause of neurologic disability in young adults [1,2]. 

The prevalence of the disease is different across the different countries: it has a high prevalence in Europe, with a north to south gradient, and a lower prevalence in Asia and Africa [3]. In Italy, we observed a disease prevalence of 176 per 100,000 inhabitants [4], except in the Mediterranean island of Sardinia, where we found an age- and sex-adjusted prevalence of MS of 330 per 100,000 inhabitants, among the highest reported worldwide, ranging from 217 in the Olbia-Tempio district to 425 in the Ogliastra district [5], with the lowest risk areas being closer to the coast.

Although most MS cases occur sporadically, about 20% of the affected individuals are related by family, with first-degree relatives of MS patients at increased risk of disease, thus suggesting that the disease is moderately heritable, with a sibling relative recurrence risk of 6.35 in the Caucasian population [6] and of 31 in the founder population of the Sardinian province of Nuoro [7].

In line with other common, complex disorders, almost 20% of risk heritability is attributable to common genetic variants in the autosomal genome, including 233 unequivocally MS-associated loci identified over the last 15 years by GWAS (genome-wide association studies), comprising 32 loci within the Major Histocompatibility Complex (MHC) [8,9,10,11,12,13], each of which explain only a small fraction of risk [14]. A recent study by the International Multiple Sclerosis Genetics Consortium (IMSGC) [15] provides evidence that 11.34% of risk heritability is explained by low-frequency variants (Minor Allele Frequency (MAF) < 5%); of these rare variants, (MAF < 1%) alone explains 9%. Most low-frequency variants impact genes that are not detectable by common variants identified by genome-wide association studies (GWAS), and only a small portion of them is in Linkage Disequilibrium (LD) with variants highlighted by GWAS.

Many low frequency and rare variant associations, as important sources of unexplained heritability, remain to be discovered [16]. This investigation would require large sample sizes to reach an appropriate statistical power, or alternatively, the use of multiplex families from a founder population for which both genotyping and sequencing data are available.

Our study aims at understanding the genetic contribution to MS and suggesting new potential causative variants in families, by contributing to the discovery of new exonic and potentially functional low-frequency variants. To this end, we analyzed multiplex families originating from the genetically homogeneous and isolated population of the Nuoro province of Sardinia for which Immunochip genotyping and whole exome-sequencing (WES) data are available. 

We followed a two-stage approach. In the first stage, we prioritized candidate regions to be further investigated via a non-parametric Homozygosity Haplotype (HH) analysis, which uses reduced haplotypes composed by homozygous single nucleotide polymorphisms (SNPs) only and deletes all the heterozygous ones. We performed this analysis on thirteen families by exploiting the co-segregation of the disease and genetic variants between affected and unaffected subjects for a genome-wide search of shared autosomal segments. In the second stage, on the promising candidate regions identified in the HH analysis, we searched for the presence of rare variants shared by the affected individuals by analyzing WES data that were available for five families only. 

## 2. Results

### 2.1. Sample Description

Thirteen multiplex Sardinian pedigrees, containing from three to sixteen MS patients each, were selected for the analysis, for a total of 80 affected (63 with Immunochip genotyping data) and 655 unaffected (220 with Immunochip genotyping data) patients.

Table 1 reports the description of the family data available for the HH analysis. We analyzed a total of 129.448 Immunochip QC-filtered SNPs that had assigned dbSNP refIDs. WES data were available for five families only. Specifically, three cases and one control for family 61, 10 cases for family 2360, two cases for family 45, four cases for family 4, and five cases for family 5.

### 2.2. Identification of RCHHs

The HH statistical analysis was performed for all the 13 families using both the genotyped patients (*n* = 63) and controls (*n* = 220), so that the algorithm worked to treat the affected and unaffected members of a family as cases and controls, respectively. As we had Immunochip data [10], obtained with an Illumina Infinium HD custom array designed for the fine mapping of 184 established autoimmune loci, and not a high-density array, we used a cutoff value of 7 cM to search the candidate RCHHs, in order to reduce the risk of false positives and to increase specificity, and we used CEUAnnotation1MDuo (http://www.hhanalysis.com/ (accessed on 15 February 2021)) as an annotation file. We selected regions with a significance level of -log_10_ (*p*-value) ≥ 1.2, corresponding to a *p*-value of 0.06, to establish difference between patient and control pools. The choice of this liberal level of significance was driven by our research strategy in which HH analysis represents a step towards prioritizing candidate regions, and thus, towards reducing false negative probability, in order to enable further investigation in the second stage (WES analysis).

**Figure 1:**
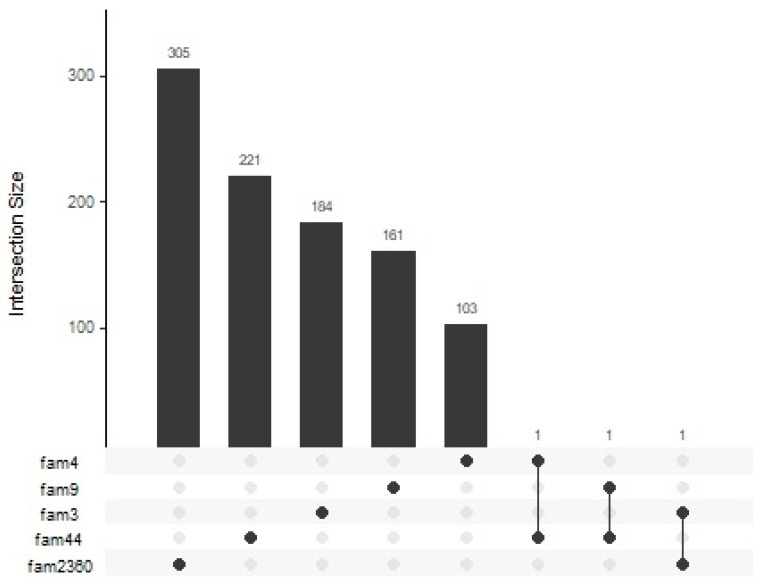
Upset plot. In the plot, the intersections of genes between families are visualized. The first five bar charts on the top of the figure represent the number of genes in the significant RCHHs found only in each individual family. The last three bar charts on the top of the figure report the number of genes shared between families.

HH was run to identify Regions with a Conserved Homozygosity Haplotype (RCHHs) that were differently shared among cases and controls (see Table 2). Then, we searched for genes in each significant RCHH, and Figure 1 reports the upset plot in which the number of genes shared between families is graphically represented. The first five bar charts on the top of the figure represent the number of genes in the significant RCHHs found only in each individual family (whose identified number is specified in the left of the figure), e.g., 305 genes that are only in family 2360 but not in the other families, etc. The last three bar charts on the top of the figure report the number of genes shared between families. In particular, families 4 and 44 share one gene, i.e., *AL833583*, and families 3 and 2360 share *DQ596042*, while families 9 and 44 share the *USP16* gene. Interestingly, increased USP16 levels, a deubiquitinase required for chromosomal segregation in mitosis [17], were observed in peripheral CD4+ T cells from patients with distinct autoimmune diseases and T cell-specific *USP16* knockout mice showed a reduced severity of experimental autoimmune encephalitis [18]. Families that were not reported in the upset plot are those who did not share any gene between them.

The Phenolyzer (http://phenolyzer.wglab.org (accessed on 30 March 2021)) and WEbGestalt (http://www.webgestalt.org (accessed on 30 March 2021)) tools were used to verify if there were genes involved in MS-related diseases due to their involvement in the CNS or immune system. We found that: (i) *CD244* (highlighted in family 44), *CIITA* (highlighted in family 44), *HLA-DRB1* (highlighted in family 2360) and *NFKBIL1* (highlighted in family 2360) are related to rheumatoid arthritis; (ii) *BANK1*, *FCGR2A*, *FCGR2B* and *FCGR2C* (highlighted in family 44) are related to systemic lupus erythematosus; and iv) *HLA-G* (highlighted in family 2360), *HNMT* (highlighted in family 44), and *TNF* (highlighted in family 2360) are related to ASMA.

### 2.3. Identification of Pathogenic Variants

**Figure 2:**
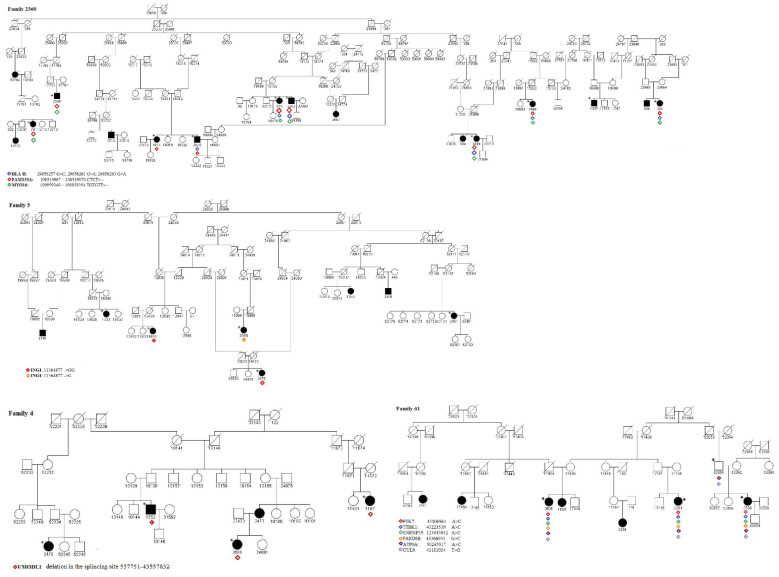
Pedigree of families in the study. Squares and circles indicate men and women, respectively. The symbols in black represent the affected members. The squares or circles with a line indicate a deceased individual. The asterisk represents subjects in the pedigree for which WES data were available, while the colored triangles indicate the subjects carrying the variant.

WES analysis of available subjects on the regions prioritized by HH analysis was performed to identify putative causative rare variants for MS. Figure 2 reports the pedigree structure of the analyzed families for which we also had WES data, and the identified variants.

From the FASTQ files, we obtained the VCF files, using bphg19 as the reference genome. The VCF files were annotated using wANNOVAR (http://wannovar.wglab.org (accessed on 3 May 2021)), and we searched for variants on the resulting csv file. Calls with synonymous variants, and benign variants were excluded, while calls with MAF reported in the 1000 genomes (https://www.internationalgenome.org/ (accessed on 10 May 2021)) dataset and gnomAD (https://gnomad.broadinstitute.org (accessed on 10 May 2021)), with values of less than 0.001 or unknown, were included. The aim was to search for rare genetic variants shared between the affected within the genes prioritized via HH analysis. 

For each family and for each RCHH, the variant, its function and the number of cases and control sharing the variant are reported in Table 3. Interestingly, three variants, i.e., 43181034 T > G and 50245517 A > C, located in splicing region on exon 19 of the *CUL9* gene and on exon 16 of the *ATAP9A* gene, respectively, and a deletion (43557751-43557832) in the splicing site of the *UMODL1* gene, were classified as photogenic in VarSome (https://varsome.com/ (accessed on 12 October 2021)). The other variants were classified as of uncertain significance.

In Table S1, for each variant, the frequency in the different populations, such as those in 1000 Genome, gnomAD, and in the Sardinian population [19,20], are reported. ST1 also reports the results obtained using different prediction algorithms, with the grade of pathogenicity for each variant.

## 3. Discussion

MS is characterized by a complex multi-factorial nature that involves the interplay of a still non-identified environmental exposure and a genetic predisposition. In recent studies, it was observed that extensive grey matter lesions in the cerebral and cerebellar cortex and hippocampus [21,22,23,24] are involved in the pathology of the disease. As with other multifactorial diseases, MS has been predominantly studied assuming the “*common disease-common variant*” paradigm via GWASs. The importance and success of the GWASs approach to identifying the loci underlying common disease cannot be overlooked [25]. These breakthroughs, along with both statistical and technological advances, have led to the identification and confirmed association of numerous genetic loci for MS susceptibility [10,11,12,26]. In spite of these advances, only a relatively small proportion of the genetic influences in MS have been uncovered, and much is yet to be understood. It is important to point out that there are alternative hypotheses concerning the genetic architecture of common diseases, including the multiple rare variant hypothesis, which may help to elucidate the so-called *missing heritability* associated with common complex diseases such as MS [27].

Our study aims at contributing to the understanding of the genetic risk component of MS and the contribution of rare variants to disease risk through a combination of HH analysis—with the aim of prioritizing candidate regions—and WES to deeply explore this candidate region in search of putative causative rare variants in the founder population of Nuoro province (Sardinia). The Sardinian population has a high prevalence of MS and autoimmune disease, and its genetic background is proven to be homogeneous and an outlier in Europe, having been under selective pressure due to malaria, which has been endemic there for centuries.

Exons are regions in the DNA that are particularly vulnerable in the presence of mutations in their sequence: an unexpected mutation may alter the structure and the function of the protein, leading to deleterious biological consequences that may contribute to—or cause—a specific disease. Thus, identifying the presence of an exonic genetic variant in a candidate region, previously highlighted by HH, will help us to better understand the biological processes involved in the disease. 

Firstly, HH analysis performed on 13 multiplex Sardinian families made it possible to identify significant RCHHs (-log_10_ (*p*-value) ≥ 1.2). Secondly, in these regions, WES data that were available for 25 subjects belonging to five families were analyzed.

Interesting results were found in:

(i) The *RCHH region on chr6:42767957-43333769*, shared in 5 cases and 7 controls of family 61, where we identified the variant 43181034 T > G in the splicing region on exon 27 of the *CUL9* gene. *CUL9* is highly expressed in the brain, particularly in the cerebral cortex [28]. A study [29] using a human cell-derived model to characterize CUL9 in human neuronal development showed that the deletion or depletion of the protein causes the aberrant formation of neural rosettes that are related to the early stage of neurodevelopment. Furthermore, the neuronal transcription factors CUX1 and SOX3 were significantly upregulated in *CUL9* knockout neuroepithelial progenitor cells. Fisher et al. [30] analyzed the potential molecular pathways of tissue injury in active cortical MS lesions, and by identifying prominent changes in gene expression, they found genes that are involved in different steps of apoptosis, DNA damage, p53 function, and DNA repair, including *CUL9*. In the same RCHH region, 43106964 A > C, a non-synonymous variant on exon 9, and rs780764712, in *PTK7*, a gene involved in the Wnt/planar cell polarity pathway, were also found. It is important to note that the *PTK7* mutant with a truncated protein perinatally caused severe defects in neural tube closure [31]. In this stidy, 43223539 A > C, a non-synonymous variant on exon 9 in the *TTBK1* gene, and 42976917 A > C, a non-synonymous variant on exon 9 in the *PPP2R5D* gene, were also found in all the available cases. TTBK1 is a brain-specific tau kinase expressed in the entorhinal cortex and hippocampal regions. *TTBK1* transgenic mice showed severe axonal degeneration in the perforant path, which is essential for many forms of memory [32]. TTBK1 is highly expressed in the entorhinal cortex and the perforant path region, two specific brain regions involved in the early stage of Alzheimer’s disease pathology [33], and thus, has a critical role in axonal degeneration. Collapsin response mediator protein-2 (CRMP2) is a downstream target of TTBK1 [32], whose expression induces the accumulation of phosphorylated CRMP2, and it was shown to be involved in the axonal degeneration pathology in MS [34]. PPP2R5D is a regulatory B subunit of Protein Phosphatase 2A (PP2A) and plays a crucial role in normal neuronal development and functioning. Variants of this gene were found to be associated with intellectual disability, autism, and other neurodevelopmental disorders [35]. Mutations in this gene were found in juvenile-onset parkinsonism [36].
(ii) The *RCHH region on chr13:108090996-108968251*, shared in 11 cases and 17 controls of family 6, where we identified the variant 109859349-109859354 TGTGTT> in 3′UTR of the *MYO16* gene. This variant is also present in 1 case of family 4, in 2 cases of family 45, and in 1 case of family 5. *MYO16* is mainly expressed in the central nervous system and seems to be involved in the development and functioning of the nervous system also in adulthood; therefore, alterations in this gene, e.g., SNPs, deletions, or epigenetic modifications, are associated with neurodegenerative and neuropsychiatric disorders [37,38,39]. *MYO16* is thus considered as an important regulator of neural cells’ functioning even if its specific role and molecular mechanisms remain to be elucidated. Interestingly, not far from *MYO16*, in the *chr13:108090996-108968251* region highlighted by HH analysis, is located the *TNFSF13B* gene, encoding the cytokine and drug target B-cell activation factor (BAFF) whose overexpression is related to autoimmunity [40]. In particular, in [41], a *TNFSF13B* variant was found to be associated with MS and systemic lupus erythematosus (SLE) through a mechanism that led to an overexpression of BAFF, which, in turn, upregulated the humoral immunity.
(iii) The *RCHH region on chr20:49044993-50323395*, shared in 5 cases and 7 controls of family 61, where we identified the variant 50245517 A > C in the splicing region on exon 16 of the *ATP9A* gene. ATP9A is a regulator of endosomal recycling and plays an inhibitory role in the release of extracellular vesicles (EV) [42], and many biological processes, such as the immune response, are modulated by proteins, DNA, miRNA, and mRNAs that could be controlled via EV-instigated intercellular communication [43].

Taken together, these results provide newer insights into the genetics of MS and a more thorough understanding of the disease biology that, in future functional studies of these highlighted specific gene variants, may provide hints towards the creation of new and more effective treatments in MS.

## 4. Materials and Methods

### 4.1. Sample Collection and Genotyping

MS patients were ascertained through the case register established in 1995 in the province of Nuoro, Sardinia, Italy. Cases were diagnosed according to Poser’s criteria [44]. Thirteen pedigrees, containing from three to sixteen MS patients each, were selected for the analysis. Genotyping data were obtained using Immunochip results obtained from a previous study [13], where the quality control-filtered dataset included 131.497 SNPs.

### 4.2. HH Analysis

HH analysis, proposed by Miyazawa et al. [45], is an efficient non-parametric tool to detect regions harboring either novel or known mutations; it makes it possible to identify patient’s shared chromosomal segments derived from a common ancestor, which are characterized by the distinct identity of their haplotype.

The analysis is based on the concept of identity-by-descent (IBD), in which a DNA segment is defined as IBD in two or more individuals if it is a direct copy of the same ancestral allele; thus, affected subjects who inherit the mutation from a common ancestor share IBD—the genetic portion around the mutation in which there should be no discordant homozygous calls for both dominant and recessive genes. HH are a reduced haplotype obtained by removing all the heterozygous SNPs from the sample dataset and leaving only the homozygous ones. The method works by comparing homozygous segments; in this way, there is no need to reconstruct haplotypes. This results in a reduced computational timing and a simplification of the analysis process, even for large numbers of SNPs. Specifically, the method makes it possible to compare the number of subjects in the patient pool and in the control pool who share an RCHH region. RCHH is a region with a given genetic length, chosen on the basis of the study, containing comparable SNPs (compSNP) in homozygosity in two subjects. The algorithm performs a pairwise comparison of individuals based on the presence of compSNP in the pair. A mismatched comparable SNP, also indicated as a discordant homozygous SNP (dhSNP), has discordant homozygous SNP genotypes in two subjects (e.g., AA vs. BB). The borders of an RCHH are defined by either dhSNPs or by the ends of the chromosome. RCHH regions shared by multiple subjects are used to predict the presence of a Region from a Common Ancestor (RCA) or an IBD [46,47]. Given m and n, the number of generations of two affected subjects descended from a common ancestor RCA is calculated as

(1) $$\;\;\;\;\;\;RCA\;\left(m,n:m\geq n\right)=\left\{\begin{array}{c} 2^{-m+1}\;\;\;\;\;\;\;\;m\geq 1,n=0\\ \frac{3}{4}\;\;\;\;\;\;\;\;\;\;\;\;m=1,n=1\\ 2^{-m-n+2}\;\;\;\;\;\;\;\;otherwise \end{array}\right\}$$

In consanguineous families or in populations that are geographically isolated, such as our Sardinia multiplex families, patients suffering from a disease (in our study, MS) share a common ancestor, and the RCA represent candidate regions in which disease genes can be looked for. RCA is identified through RCHHs via the comparison of RCHHs shared between the patient and the control pool [45]. As explained in [46], the algorithm works firstly by dividing a genetic autosomal region into smaller ones and selecting, for each of them, the representative RCHHs shared by the largest number of affected subjects within the patient pool. The numbers of individuals sharing each representative RCHH pool are then counted in both the patient and the control groups and compared to each other. The *p*-value of the null hypothesis of no difference between these two proportions is calculated according to the standard normal distribution.

In the HH approach, genotyping error may lead to be causative gene being excluded mistakenly. In fact, an RCHH will be truncated when a non-dhSNP changes into a dhSNP due to a genotyping error; in this way, the resulting genomic segment will be smaller than the chosen cut-off length, and thus, the RCHH will be not identified. The algorithm, by means of Monte Carlo Chain Simulation (MCMC), makes it possible to calculate the probability of obtaining dhSNPs due to genotyping errors. If the probability of creating dhSNPs in the region is very low (*p*-value < 0.001), the HH results are reliable.

A critical step in the analysis is represented by the choice of cut-off value since there is not an optimal cut-off value. They need to be chosen on the basis of the population under study, taking into account that the decreases in the average genetic length of the RCAs over generations; therefore, when we analysed distantly related subjects who shared smaller RCA, it was preferable to use a small cut-off (e.g., 3 cM). Another aspect that guides our choice of the cut-off is the array used for genotyping (e.g., low-density vs. high-density). As we only had Immunochip data [10], which were obtained with the Illumina Infinium HD custom array that was designed for the fine mapping of 184 established autoimmune loci, we chose a conservative cutoff value of 7 cm to search for candidate RCHHs that represent our prioritized regions. This step was taken to reduce the risk of false positives and increase the specificity of the results.

In summary, the advantages of HH approach numerous are: (a) there is no need to reconstruct haplotypes since the homozygote haplotype for each chromosome is uniquely determined; (b) the chromosomal segments in which all polymorphic markers are homozygous are considered to be autozygous segments [48]; (c) if the coefficient of consanguinity for a patient is large as a result of belonging to an inbred family, and the disease is rare, then the probability that the disease-causing gene is located in the shared segment is very high; (d) since HH analysis looks for ancestral segments, both dominant and recessive genes can be detected; (e) the HH approach is robust to genotyping errors.

In our analysis, the dataset was prepared using R software [49], and the HH analysis was run using the HH program (http://www.hhanalysis.com (accessed on 15 February 2021)).

### 4.3. Screening of Known Causative Genes

We then scrutinized the segments highlighted in the HH analysis with the help of publicly available databases such as Phenolyzer (http://phenolyzer.wglab.org (accessed on 30 March 2021)) and WEbGestalt (http://www.webgestalt.org (accessed on 30 March 2021)), in order to search for genes located, within the specified region, whose function could be plausibly related to MS.

### 4.4. Whole-Exome Sequencing Data Generation

All samples were sequenced at the Center for Genome Technology within the University of Miami John P. Hussman Institute for Human Genomics.

Library preparation was conducted using the SureSelectXT Human All Exon V4 + UTR (Agilent Technologies Inc., Santa Clara, CA, USA). This protocol targets 99% of coding regions in addition to 5′ and 3′-untranslated region sequences. Pre-enrichment libraries were constructed using the SureSelect Low Input reagent kit, and exome enrichment of the DNA library was performed via a hybridization reaction with biotinylated baits from the SureSelect Human All Exon V4 + UTR Enrichment Kit. Sequencing of the prepared DNA libraries was undertaken using the Illumina HiSeq2000 instrument (Illumina Inc., San Diego, CA, USA) with an average coverage of 80× with 2 × 100 bps paired-end reads. Quality controls were applied at the lane and fastq levels. Specifically, the cutoff used for a successful lane was Pass Filter > 90%, with over 250 M reads for the high-output mode. The fraction of reads in each lane assigned to each sample (no set value) and the fraction of bases with a quality score > Q30 for read 1 and read 2 (above 80% expected for each) were also checked. Raw sequencing reads were demultiplexed using Illumina bcl2fastq. In addition, the FASTQC tool kit (www.bioinformatics.babraham.ac.uk/projects/fastqc/ (accessed on 8 November 2020)) was used to review the base quality distribution, which provided representations of the four nucleotides of particular k-mer sequences (adaptor contamination). We used the Genome Analysis Software Kit (GATK) (version 4.1) best-practice pipeline to analyze our WES data. Reads were aligned with the human reference genome (hg19), using the Maximal Exact Matches algorithm in the Burrows–Wheeler Aligner (BWA) [50]. PCR duplicates were removed using the Picard tool (picard.sourceforge.net/). The GATK base quality score recalibrator was applied to correct the sequencing artifacts. Variants were called using the GATK haplotypeCaller algorithm, visually inspected using the Integrative Genomics Viewer (IGV, Broad Institute), and further annotated with ANNOVAR. Variants were categorized as follows: (1) non-synonymous; (2) synonymous; (3) frameshift deletion or insertion; (4) splicing; (5) stop gain or loss; or (6) functional intronic or promoter variants.

## 5. Conclusions

Our study, which was performed on multiplex Sardinian families and combined the HH approach with WES data analysis, first enabled the identification of disease-linked regions, and then the identification of specific rare variants located in these regions. Although our study was limited to the use of Immunochip data that, despite making it possible to scrutinize the entire genome, only allowed this within specific autoimmune candidate regions, the obtained results represent an important step in the comprehension of the genetics of MS.

## Figures and Tables

**Table 1:** Description of the family data available for the HH analysis. For each family, the total numbers of affected and unaffected subjects are reported together with the availability of Immunochip genetic data.

Family	Total N. of Affected	N. of Affected with Genotyping Data	Total N. of Unaffected	N. of Unaffected with Genotyping Data
3	6	5	84	29
4	5	5	37	23
5	8	5	79	22
9	9	8	64	22
12	3	3	19	3
21	5	5	53	19
26	6	3	43	13
44	3	3	14	9
45	6	4	36	15
58	3	2	27	14
61	7	6	40	17
81	3	3	23	8
2360	16	11	136	26

**Table 2:** List of RCHHs grouped by family. The table reports the number of subjects sharing an RCHH in both pools, its coordinate and the -log_10_ (*p*-value) for the difference between the pools. In our study, we set a significance level of -log_10_ (*p*-value) ≥ 1.2, corresponding to a *p*-value of 0.06.

Family	No. Subjects Sharing RCHH in Patient Pool	No. Subjects Sharing RCHH in Control Pool	Chr	Start–End (bp *)	Start–End (SNP)	-log_10_(*p*-Value)
2360	6 out of 11	7 out of 26	1	20024018-20110347	rs12405947-rs10492999	1.20
2360	8 out of 11	11 out of 26	3	127665610-128219449	rs1687462-rs9826526	1.23
2360	5 out of 11	5 out of 26	6	26287459-32628250	rs4458680-rs11757159	1.25
2360	7 out of 11	9 out of 26	11	16548170-17019030	rs4593976-rs7942085	1.20
2360	11 out of 11	19 out of 26	13	106416156-106713791	rs9555302-rs1819243	1.30
2360	11 out of 11	18 out of 26	13	106716741-106978764	rs3949948-rs16970623	1.47
2360	11 out of 11	19 out of 26	13	106979630-106979630	rs1830754-rs1830754	1.30
2360	11 out of 11	18 out of 26	13	106979661-108046934	rs2076766-rs7992149	1.47
2360	11 out of 11	19 out of 26	13	108048315-108048315	rs9583266-rs9583266	1.30
2360	11 out of 11	18 out of 26	13	108050353-108090301	rs12586075-rs16972849	1.47
2360	11 out of 11	17 out of 26	13	108090996-108968251	rs16972855-rs9521415	1.63
2360	7 out of 11	9 out of 26	22	43217334-43366873	rs133807-rs138628	1.20
61	5 out of 6	7 out of 17	2	221458760-223560597	rs634813-rs1440063	1.22
61	6 out of 6	10 out of 17	4	181036139-181598064	rs7655585-rs2727426	1.23
61	6 out of 6	10 out of 17	5	31060610-31060610	rs1392428-rs1392428	1.23
61	5 out of 6	7 out of 17	6	42767957-43333769	rs394754-rs7752120	1.22
61	5 out of 6	7 out of 17	6	45282619-46437363	rs7762957-rs1372567	1.22
61	4 out of 6	4 out of 17	6	62416537-67861137	rs213824-rs9294736	1.38
61	4 out of 6	4 out of 17	6	100591693-100883678	rs9399393-rs2658132	1.38
61	5 out of 6	7 out of 17	7	8552281-8768564	rs1859275-rs10268580	1.22
61	6 out of 6	9 out of 17	9	8991207-8991239	rs10511519-rs10816028	1.41
61	6 out of 6	8 out of 17	9	8991538-8994896	rs10511520-rs10816029	1.61
61	6 out of 6	9 out of 17	9	9000336-9395003	rs7025315-rs10759064	1.41
61	6 out of 6	10 out of 17	9	9395316-11328859	rs1475680-rs17788370	1.23
61	6 out of 6	10 out of 17	9	12662320-13015284	rs1408801-rs10514822	1.23
61	5 out of 6	7 out of 17	9	115676064-115712622	rs6478042-rs7034929	1.22
61	6 out of 6	10 out of 17	9	134997809-134997809	rs626713-rs626713	1.23
61	6 out of 6	10 out of 17	12	122479650-122743242	rs1706477-rs7137946	1.23
61	6 out of 6	10 out of 17	12	127998742-128288581	rs11060036-rs10847807	1.23
61	5 out of 6	7 out of 17	16	84533519-84542101	rs305059-rs908988	1.22
61	5 out of 6	6 out of 17	20	48202462-49044808	rs6020298-rs6122991	1.43
61	5 out of 6	7 out of 17	20	49044993-50323395	rs11904901-rs6068117	1.22
61	5 out of 6	6 out of 17	20	50326580-50563902	rs6021835-rs2024650	1.43
45	4 out of 4	7 out of 15	1	22014570-23032859	rs2010397-rs4654821	1.22
45	3 out of 4	4 out of 15	7	54280160-56646646	rs13438238-rs2634081	1.21
45	4 out of 4	7 out of 15	13	72551882-73411123	rs4883922-rs728926	1.22
45	4 out of 4	6 out of 15	13	73411181-75116077	rs17288193-rs1006412	1.40
44	3 out of 3	3 out of 9	1	2289487-2455004	rs7545940-rs9803764	1.24
44	3 out of 3	3 out of 9	1	156653770-160351933	rs2188102-rs1415259	1.24
44	3 out of 3	3 out of 9	2	138041133-138458405	rs7563139-rs10199542	1.24
44	3 out of 3	3 out of 9	4	96765575-103179634	rs11941922-rs2129294	1.24
44	3 out of 3	3 out of 9	5	73747-556484	rs7709758-rs6420045	1.24
44	3 out of 3	3 out of 9	5	150483977-150816773	rs2303027-rs17802828	1.24
44	3 out of 3	3 out of 9	5	154874578-156864954	rs1295243-rs2277027	1.24
44	3 out of 3	3 out of 9	7	142903019-142909027	rs6963381-rs1880560	1.24
44	3 out of 3	3 out of 9	8	602758-3038563	rs9314595-rs13261550	1.24
44	3 out of 3	3 out of 9	16	10909415-11580966	rs2229321-rs8050461	1.24
26	3 out of 3	3 out of 13	1	171619320-173691804	rs6701066-rs860905	1.65
26	3 out of 3	4 out of 13	1	173695580-174106457	rs1016815-rs10798418	1.40
26	3 out of 3	4 out of 13	14	80069595-82872217	rs1543918-rs17625929	1.40
21	5 out of 5	10 out of 19	1	14226898-14226898	rs4579751-rs4579751	1.27
21	5 out of 5	10 out of 19	5	73478194-73598034	rs2120729-rs1460812	1.27
9	4 out of 8	4 out of 22	11	45356859-51450167	rs717653-rs12291581	1.31
9	4 out of 8	4 out of 22	12	44020977-44336606	rs10785572-rs878111	1.31
9	7 out of 8	10 out of 22	21	27810551-29354016	rs2830992-rs1064019	1.48
5	3 out of 5	4 out of 22	5	58726008-59841224	rs525099-rs40512	1.41
5	4 out of 5	8 out of 22	9	85066453-85227053	rs10867967-rs871790	1.21
5	4 out of 5	7 out of 22	9	85228118-85448505	rs3860918-rs1052690	1.39
5	5 out of 5	12 out of 22	13	109106993-109251608	rs9515092-rs7986346	1.22
5	5 out of 5	11 out of 22	13	109253434-109607383	rs11069806-rs9521623	1.36
5	4 out of 5	8 out of 22	13	109945232-110218960	rs9555712-rs12865465	1.21
4	5 out of 5	12 out of 23	1	237070145-237394008	rs869035-rs1980004	1.30
4	5 out of 5	11 out of 23	1	237395275-238575190	rs11808376-rs10495466	1.43
4	5 out of 5	12 out of 23	1	238576784-238576784	rs7552602-rs7552602	1.30
4	5 out of 5	11 out of 23	1	238579605-238713399	rs12137050-rs9662136	1.43
4	5 out of 5	11 out of 23	7	142336895-143056687	rs4236481-rs12540188	1.43
4	5 out of 5	12 out of 23	7	143059971-143855577	rs4640977-rs2057868	1.30
4	5 out of 5	11 out of 23	7	143858588-144083589	rs17169930-rs7793227	1.43
4	5 out of 5	12 out of 23	7	144088382-145211344	rs6954142-rs4601231	1.30
4	5 out of 5	12 out of 23	8	126600646-126663920	rs4006563-rs7016867	1.30
4	5 out of 5	12 out of 23	8	126672376-128144872	rs4870946-rs1456314	1.30
4	5 out of 5	12 out of 23	13	109370211-109416832	rs7323507-rs7984646	1.30
4	5 out of 5	11 out of 23	13	109419064-109607383	rs9583447-rs9521623	1.43
4	5 out of 5	11 out of 23	14	92683815-94120061	rs12589195-rs12880862	1.43
4	5 out of 5	12 out of 23	14	94122073-94216494	rs2069956-rs7148204	1.30
4	5 out of 5	12 out of 23	17	67527147-67755206	rs9914764-rs12939271	1.30
4	5 out of 5	12 out of 23	21	42351204-42525479	rs17114247-rs881395	1.30
4	5 out of 5	11 out of 23	21	42525851-42616108	rs915846-rs691567	1.43
4	5 out of 5	12 out of 23	22	24549605-24559502	rs4820658-rs4822661	1.30
4	5 out of 5	12 out of 23	22	24561312-24579927	rs17704912-rs2748234	1.30
3	3 out of 5	6 out of 29	1	171432870-171774723	rs1234313-rs1461019	1.30
3	1 out of 5	1 out of 29	2	90959860-91680834	rs10201040-rs4373803	1.23
3	5 out of 5	14 out of 29	3	71749225-72031216	rs864380-rs9861583	1.38
3	5 out of 5	15 out of 29	3	72031489-72458673	rs1995453-rs4303823	1.27
3	5 out of 5	14 out of 29	3	72459498-74148704	rs6790069-rs1405396	1.38
3	5 out of 5	14 out of 29	3	74710393-74710393	rs13073838-rs13073838	1.38
3	4 out of 5	10 out of 29	3	75820822-76074141	rs536575-rs4095546	1.27
3	1 out of 5	1 out of 29	5	69782071-69967168	rs169717-rs3871460	1.23
3	4 out of 5	10 out of 29	6	52061483-52205031	rs6906409-rs6913472	1.27
3	4 out of 5	9 out of 29	6	52205660-52213626	rs9395771-rs9382084	1.42
3	5 out of 5	10 out of 29	7	4275511-4316475	rs10272180-rs2107834	1.96
3	5 out of 5	11 out of 29	7	4318943-4930528	rs2097884-rs2089967	1.79
3	5 out of 5	12 out of 29	7	4930906-4930906	rs6947947-rs6947947	1.64
3	5 out of 5	11 out of 29	7	4934189-5342068	rs13224720-rs10234709	1.79
3	5 out of 5	12 out of 29	7	5344404-5549582	rs13238999-rs2098225	1.64
3	5 out of 5	13 out of 29	7	5551125-6459404	rs1725213-rs7810553	1.50
3	5 out of 5	14 out of 29	7	6461542-7642706	rs7792987-rs10280185	1.38
3	5 out of 5	15 out of 29	7	7767212-7831276	rs17137412-rs12702661	1.27
3	5 out of 5	15 out of 29	7	150524562-150524562	rs310586-rs310586	1.27
3	5 out of 5	14 out of 29	7	150526978-150611097	rs7458773-rs6953552	1.38
3	5 out of 5	15 out of 29	7	150613053-150619105	rs7797007-rs219245	1.27
3	1 out of 5	1 out of 29	9	40059290-44362584	rs375972-rs4929023	1.23
3	1 out of 5	1 out of 29	9	44670536-46992793	rs12006135-rs7049015	1.23
3	1 out of 5	1 out of 29	9	65231255-65635106	rs28533023-rs1480368	1.23
3	1 out of 5	1 out of 29	13	111553045-114123122	rs12017986-rs12874290	1.23
3	1 out of 5	1 out of 29	20	28039018-28259678	rs7267880-rs6567465	1.23

* SNP Position hg18.

**Table 3:** Results of WES analysis. For each family and for each RCHH region, identified by HH analysis, the variant, its function, and the number of cases and controls sharing the variant are reported.

Family	RCHH Region (bph18)	Chr	Start	End	Ref	Alt	Function	Gene	No. of Affected	No. of Unaffected
61	chr6: 42767957-43333769	6	43181034	43181034	T	G	splicing region on exon 27	*CUL9*	3 out of 3	1 out of 1
		6	43106964	43106964	A	C	non-synonymous variant on exon 9	*PTK7*	3 out of 3	0 out of 1
		6	43223539	43223539	A	C	non-synonymous variant on exon 9	*TTBK1*	3 out of 3	0 out of 1
		6	42976917	42976917	A	C	non-synonymous variant on exon 9	*PPP2R5D*	3 out of 3	0 out of 1
	chr20:48202462-49044808	20	49366933	49366933	G	C	non-synonymous variant on exon 3	*PARD6B*	3 out of 3	0 out of 1
	chr20:49044993-50323395	20	50245517	50245517	A	C	splicing region on exon 16	*ATP9A*	3 out of 3	1 out of 1
	chr12:122479650-122743242	12	123943942	123943942	A	C	intronic variant	*SNRNP35*	3 out of 3	0 out of 1
	chr2:221458760-223560597	2	223554057	223554057	T	G	non-synonymous variant on exon 3	*MOGAT1*	3 out of 3	1 out of 1
2360	chr6:26287459-32628250	6	29856257	29856257	G	C	non-coding RNA	*HLA-H*	6 out of 10	NA
		6	29856261	29856261	G	A	non-coding RNA	*HLA-H*	6 out of 10	NA
		6	29856263	29856263	G	A	non-coding RNA	*HLA-H*	6 out of 10	NA
	chr13:108090996-108968251	13	108519067	108519070	CTCT	-	5′UTR	*FAM155A (NLF-1)*	9 out of 10	NA
		13	109859349	109859354	TGTGTT	-	3′UTR	*MYO16*	7 out of 10	NA
4	chr21:42351204-42525479	21	43557751	43557832			deletion on slicing site	*UMODL1*	3 out of 4	NA
5	chr13:109945232-110218960	13	11364877	11364877	-	GGG	insertion in the upstream site	*ING1*	3 out of 5	NA

NA = No WES data were available for unaffected subjects in the family.

## Data Availability

Data can be found here https://doi.org/10.5281/zenodo.5493638 (accessed on 8 September 2021).

## Supplementary Materials

The following are available online at https://www.mdpi.com/article/10.3390/cimb43030125/s1.
